# Machine learning for optimizing mAs in KUB radiography with metal implants

**DOI:** 10.1002/acm2.70493

**Published:** 2026-01-30

**Authors:** Wen‐Xuan Chen, Jen‐Pei Su, Shih‑Hua Huang, Sin‑Rong Huang, Ming‐Chung Chou

**Affiliations:** ^1^ Department of Medical Imaging and Radiological Sciences Kaohsiung Medical University Kaohsiung Taiwan; ^2^ Department of Medical Imaging Kaohsiung Medical University Hospital Kaohsiung Taiwan; ^3^ Department of Medical Imaging E‑Da Hospital Kaohsiung Taiwan; ^4^ Department of Interventional Cardiology E‑Da Hospital Kaohsiung Taiwan; ^5^ Biomedical Artificial Intelligence Academy Kaohsiung Medical University Kaohsiung Taiwan; ^6^ Center for Big Data Research Kaohsiung Medical University Kaohsiung Taiwan; ^7^ Department of Medical Research Kaohsiung Medical University Hospital Kaohsiung Taiwan

**Keywords:** KUB radiography, machine learning, mAs, target exposure indicator

## Abstract

**Background and purpose:**

Kidney–ureter–bladder (KUB) radiography is a common examination that exposes patients to a higher radiation dose and increased cancer risk; therefore, it is important to estimate suitable exposure factors for each patient prior to radiography. The present study aimed to utilize machine learning (ML) approach to predicting the suitable milliampere‐seconds (mAs) and reducing overexposure in patients with metal implants during KUB radiography.

**Methods:**

A phantom was used to understand the effect of metal implants on radiation exposure during KUB radiography with automatic exposure control (AEC) technique. Subsequently, we retrospectively enrolled 619 subjects, including 56 with metal implants and 563 without, from one hospital (group A) and 323 subjects, including 89 with metal implants and 234 without, from another hospital (group B). All subjects underwent both KUB radiography and physiological examinations on the same day. Data on body parameters and exposure factors were retrieved from hospital database. To train the prediction model, the dataset of group A without metal implants was randomly divided into 80% and 20% for training and testing sets, respectively. Five different ML algorithms were utilized to train the prediction model using 10‐fold cross‐validation. The correlation coefficients (CC), mean average error (MAE), normalized root mean squared errors (nRMSE), and R‐square (R^2^) were compared to find the optimal model. For external validation, the dataset of group B was randomly separated into 80% and 20% for training and testing sets, respectively. The training sets of both groups were combined for transfer learning, and the testing set of the group B was used to assess the optimal model. Furthermore, the final model was utilized to predict an appropriate mAs for patients with metal implants in both groups. Statistical analysis was performed to understand the differences between datasets, phantom settings, and ML models. Comparisons were considered significance if *p* < 0.05.

**Results:**

The phantom experiment demonstrated that the metal plate significantly increased the mAs and reached exposure (REX) values when using AEC technique during KUB radiography. The comparison of patient data showed that the patients with metal implants had significantly higher mAs and REX than those without in both groups. In group A, the ML comparisons showed that the artificial neural network (ANN) model outperformed other ML models in predicting mAs based on the testing set, exhibiting the highest CC of 0.791 ± 0.007 and *R* ^2^ of 0.6193 ± 0.010. In group B, the external validation based on transfer learning demonstrated that the ANN model achieved the CC of 0.837 ± 0.051 and *R* ^2^ of 0.823 ± 0.007 in the testing set. For patients with metal implants, the ANN model‐predicted mAs was significantly lower than those obtained using AEC technique in both groups.

**Conclusion:**

We concluded that the ML approach is suitable for building the model for predicting appropriate mAs and reducing overexposure in patients with metal implants during KUB radiography.

## 1 INTRODUCTION

Kidney–ureter–bladder (KUB) radiography is a diagnostic procedure used to examine the urinary system and abdominal organs, including the kidneys, ureters, and bladder. It is commonly employed as a first‐line diagnostic tool for patients experiencing abdominopelvic pain, suspected urinary stones or calcifications, gastrointestinal blockages, or malignancies. The KUB examination encompasses the abdominal and pelvic areas, extending from the kidneys to the symphysis pubis, with its center at the level of the bilateral iliac crests. However, patients with urolithiasis may undergo repeated imaging during follow‐up, which increases their risk of higher level of radiation exposure compared to other types of abdominal radiography.^1^ Besides, the entrance skin dose to patients during KUB radiography was very high among all x‐ray examinations,^2^ so incorrect exposure settings may result in very high risk of overexposure. A previous study showed that the mean effective dose of KUB radiography is comparable to that of low‐dose computed tomography for patients with a body mass index (BMI) greater than 35 kg/m^2^.^3^ Therefore, careful adjustment of exposure techniques is essential to avoid both insufficient and excessive exposure for patients undergoing KUB radiography.

In radiography, image quality is influenced by both exposure parameters, such as kilovoltage peak (kVp) and milliampere‐seconds (mAs), as well as body parameters, including the patient's weight (W), height (H), and BMI.^4^, ^5^, ^6^, ^7^, ^8^, ^9^, ^10^ Additionally, image quality is related to the performance of radiographic systems, including detector sensitivity, post‐processing pipelines, and tube anode angles.^11^, ^12^, ^13^, ^14^ In clinical practice, radiation technologists or radiographers typically select appropriate exposure parameters and set an upper limit for mAs based on the patients' body weights and heights when utilizing the automatic exposure control (AEC) technique. However, it was demonstrated that using AEC technique may lead to increased radiation doses due to variations in patient positioning or the presence of metal implants.^15^, ^16^ Therefore, it is essential to accurately determine the exposure parameters and the upper limit of the mAs to ensure consistent image quality.

Quantitative measurement of radiographic image quality can be achieved through exposure indicators that exhibit a linear relationship with detector exposure, such as exposure index (EI) and reached exposure (REX).^17^, ^18^, ^19^, ^20^, ^21^, ^22^ In clinical practice, REX operationally relates to EI by serving as a standardized metric to ensure that the exposure is within optimal ranges for image quality while minimizing patient dose. Given the linear or inverse linear correlation between image quality and exposure indicators, previous studies have recommended determining a target exposure index (EI_T_) specific to different anatomical examinations to ensure consistent image quality.^18^, ^20^ By comparing the disparity between the actual exposure indicators and the EI_T_, one can objectively assess the level of image quality achieved during radiography. In this regard, a previous study proposed the recommended EI_T_ value obtained by averaging the REX values from the repeated phantom radiographic procedures.^20^

A recent study utilized machine learning (ML) to develop a predictive model for estimating the appropriate mAs for digital chest radiography and used anthropomorphic phantom to estimate target REX in the prediction model.^23^ However, the physical properties of the materials in the phantom differ from those in human tissue, so the EI_T_ measured from the phantom may not be suitable for human studies. Although the previous study demonstrated that the ML model could accurately predict suitable mAs for chest radiography in patients without metal implants, it remains uncertain whether this predictive model can effectively mitigate overexposure in patients with metal implants during radiography. Given that metal implants can significantly absorb incident x‐ray photons, it is anticipated that fewer photons will reach the AEC sensor, potentially resulting in overexposure for these patients. To our knowledge, no previous study has created a predictive model specifically aimed at estimating appropriate mAs values to reduce overexposure in patients with metal implants during KUB radiography. Therefore, the goals of this study were to demonstrate the effect of metal implants on radiation exposure using phantom experiment, to develop a predictive model for determining the appropriate mAs settings during KUB radiography, and to apply this model to minimize overexposure in patients with metal implants.

## 2 MATERIALS AND METHODS

### 2.1 Phantom study

**FIGURE 1:**
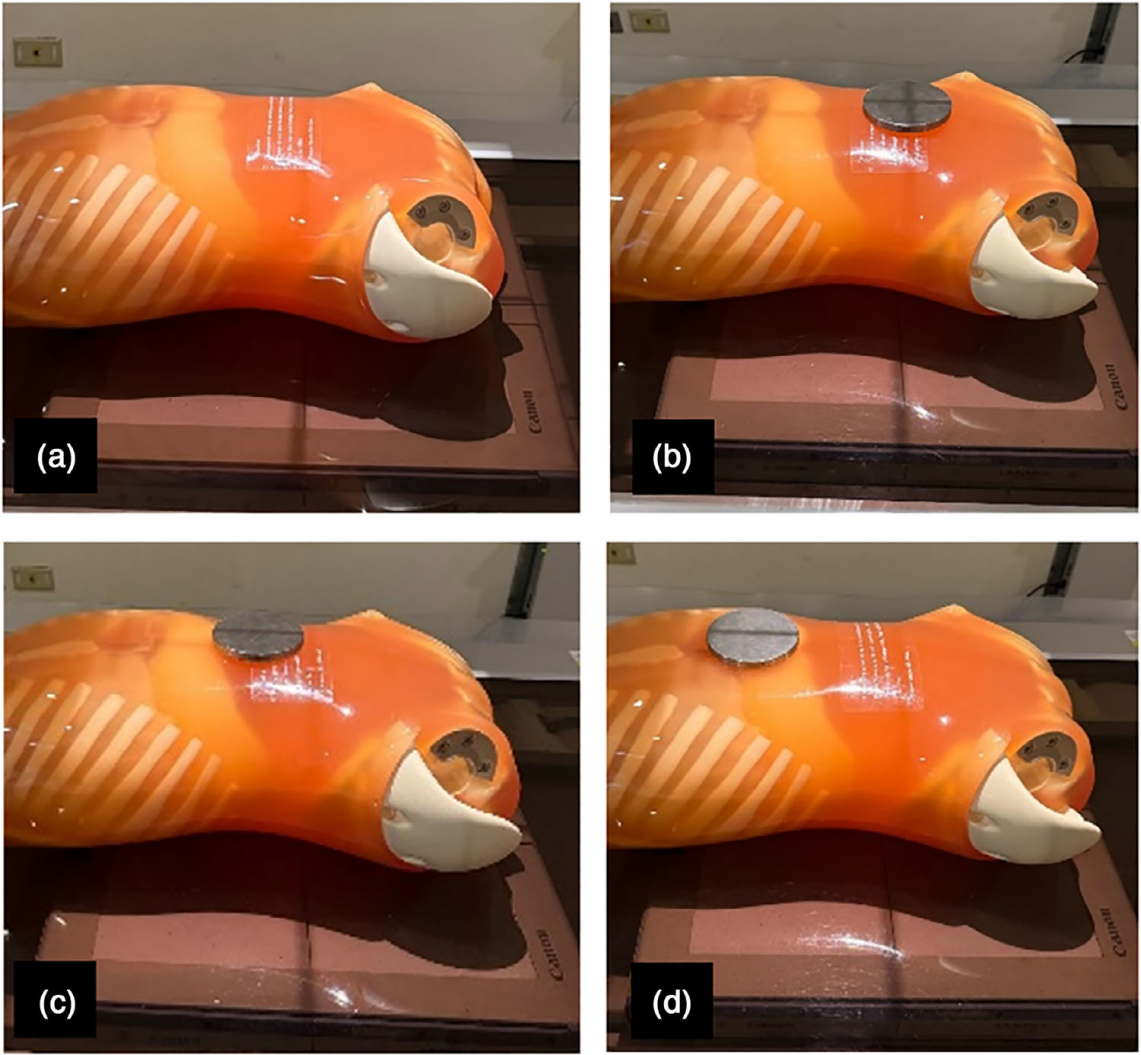
The anthropomorphic phantom was positioned for KUB radiography (a) without a metal plate, (b) with a metal plate placed at the center, (c) 5 cm away from the center, and (d) the edge of field of view.

An anthropomorphic phantom (Kyoto Kagaku, Japan) was centrally positioned within the field of view (FOV) and subjected to repeated exposures to obtain ten images using a digital x‐ray imaging system (CXDI‐70C CANON; FPD‐TFT; KXO‐A50S TOSHIBA). As the phantom represents a normal sized human with height and weight of 165 cm and 50 kg, respectively, the present study used an exposure factor of 74 kVp and the AEC technique was utilized to derive the mAs with all AEC chambers being turned on. The source‐to‐image distance was 100 cm and the FOV was 14 × 17 inches to cover entire flat panel detector. To understand the impact of metal implants on radiation exposure when using the AEC technique, an iron plate with a diameter of 10 cm and a thickness of 1 cm was placed on the phantom at three locations: at the center, 5 cm from the center, and at the edge of the FOV during KUB radiography, as illustrated in Figure 1. The iron plate was chosen to represent high‐Z attenuation effects during radiography.

### 2.2 Patient study

This study was approved by the local institutional review board of Kaohsiung Medical University Hospital (protocol code: KMUHIRB‐E(I)‐20200459) and E‐Da Hospital (protocol code: EMRP‐109‐165). The research was conducted in accordance with the Helsinki Declaration, and the informed consent was waived due to the retrospective nature of the study. From January 2017 to December 2019, 619 subjects (309 males and 310 females) of Kaohsiung Medical University Hospital (group A) and 323 subjects (162 males and 161 females) of E‐Da Hospital (group B) who underwent both anterior‐to‐posterior supine KUB radiography and physiological examinations on the same day were retrospectively enrolled. The inclusion criteria specified that patients were aged ≥ 18‐years‐old and must have undergone routine supine KUB radiography, with their W, H, and BMI recorded in our hospital information system database. However, those patients who underwent KUB radiography with incomplete physiological data were excluded from the study. Additionally, the kVp, mAs, and corresponding REX values were extracted from the picture archiving and communication system (PACS) database by analyzing the digital imaging and communication in medicine (DICOM) header of the images acquired from two independent digital x‐ray imaging systems (KXO‐A50S TOSHIBA and KXO‐80SS TOSHIBA) in respective hospitals. Two x‐ray imaging systems have periodically undergone routine quality control program to maintain consistent imaging performance. Furthermore, all radiographs were reviewed by radiologists and demonstrated adequate diagnostic image quality. Table 1 shows the demographic characteristics of two patient groups enrolled from two hospitals.

**TABLE 1:** The demographic characteristics of two patient groups. Data are expressed as mean ± standard deviation. Asterisks (*) indicate significant difference between patients with and without metal implants (*p* < 0.05).

Group A	Age (years)	Weight (kg)	Height (cm)	BMI (kg/m^2^)	W/H (kg/m)	kVp	mAs	REX
With metal implants (M/F = 25/31)	69.7 ± 14.0*	67.9 ± 18.3	158.5 ± 13.6	25.8 ± 4.5	0.45 ± 0.26	77.3 ± 3.3	92.1 ± 35.6*	758.8 ± 128.5*
Without metal implants (M/F = 284/279)	55.4 ± 18.8*	67.0 ± 27.0	162.4 ± 9.1	25.6 ± 11.5	0.41 ± 0.16	76.5 ± 2.7	79.6 ± 34.4*	716.3 ± 118.0*

Group B	**Age (years)**	**Weight (kg)**	**Height (cm)**	**BMI (kg/m^2^)**	**W/H (kg/m)**	**kVp**	**mAs**	**REX**
With metal implants (M/F = 34/55)	61.0 ± 12.9	63.8 ± 10.2	160.2 ± 7.9	24.8 ± 3.7	0.40 ± 0.06	85.0 ± 0.0	35.6 ± 13.2*	618.5 ± 74.7*
Without metal implants (M/F = 128/106)	58.0 ± 16.9	62.9 ± 12.6	161.6 ± 8.6	24.0 ± 3.9	0.39 ± 0.07	85.0 ± 0.0	29.9 ± 12.5*	547.9 ± 73.9*

*Note*: Metal implants refer to surgical devices, such as orthopedic implants, vascular stents, spinal implants, cardiac devices, and others, and appear in the image. Abbreviations: BMI = body mass index; H = height; kVp = peak kilovoltage; mAs = milliampere seconds; REX = reached exposure; W = weight.

### 2.3 Machine learning

In order to train a predictive model, this study focused exclusively on a dataset of patients without metal implants, based on evidence suggesting that metal implants may contribute to overexposure when using the AEC technique.^15^, ^16^ In group A, the patients without metal implants were then divided into 80% training and 20% testing subsets through a semi‐random selection process that ensured a random match of patients' demographic characteristics, as shown in Table 2. In the present study, age, sex, kVp, W, H, W/H, BMI, and REX values without normalization were used as input parameters to train the mAs prediction model. Five different ML algorithms were employed to develop the predictive model, including support vector machine (SVM), random forest (RF), bagging aggregation (BAG), artificial neural network (ANN), and least absolute shrinkage and selection operator (LASSO), which were commonly used for regression problem.^23^, ^24^ Moreover, since there are different kernel functions for SVM algorithm (linear, Gaussian, and polynomial) and various subtypes for ANN algorithm (CasscadeForwardNet, ElmanNet, FeedForwardNet, and FitNet), the present study only selected the best SVM and ANN prediction models for comparison.

**TABLE 2:** The training and testing datasets for patients without metal implants of group A. Data are expressed as mean ± standard deviation. Note that no significant difference was noted between the training and testing sets.

Group A	Age (years)	Weight (kg)	Height (cm)	BMI (kg/m^2^)	W/H (kg/m)	kVp	mAs	REX
Training set (M/F = 235/227)	55.5 ± 20.1	67.2 ± 29.3	162.3 ± 9.1	25.7 ± 12.6	0.47 ± 0.17	76.6 ± 2.8	79.0 ± 34.3	715.7 ± 118.7
Testing set (M/F = 49/52)	54.7 ± 18.3	66.0 ± 11.8	162.6 ± 8.7	24.9 ± 3.9	0.41 ± 0.06	76.4 ± 2.2	82.4 ± 35.0	719.3 ± 115.3

Abbreviations: BMI = body mass index; H = height; kVp = peak kilovoltage; mAs = milliampere second; REX = reached exposure; W = weight.

For SVM models, an appropriate scale factor was selected using a heuristic procedure, and no hyperparameter optimization was performed to avoid a risk of overfitting during the training. For ANN models, two hidden layers with a size of ten neurons were designed to construct the model because only eight variables were used as predictors. For LASSO, the regularization parameter (*λ*) was 100, and *λ* ratio was 0.0001 during the training. For BAG, the number of learning cycles was 100, and no hyperparameter optimization was performed. For RF, number of trees was 100, and no hyperparameter optimization was performed during model training. Moreover, no feature selection was performed in this study because only eight variables were used as model predictors. All ML models were trained using 10‐fold cross‐validation, and the testing set was used to assess their performance using the best models. The normalized root mean squared errors (nRMSE), R‐square (R^2^), mean average error (MAE), and correlation coefficients (CC) between the model‐predicted and actual AEC‐derived values were utilized to compare the performance of ML models. Finally, the feature importance of the best model was evaluated using an out‐of‐bag permutation test conducted 1000 times.

### 2.4 Transfer learning

To test the model generalizability, the built model was externally validated based on transfer learning using the dataset of patient group B collected from the other hospital. The group B used a fixed 85 kVp to ensure consistent imaging condition, and the difference of kVp between the two groups could help understand the model performance under different exposure settings. In group B, 80% training set and 20% testing set were randomly separated using a semi‐random selection that ensured a random match of patients' demographic characteristics, as shown in Table 3. For transfer learning, the training sets of two groups were integrated to train the model, and the testing set of the group B was used for external validation. After 10‐fold cross validation, the nRMSE, R‐square, MAE, and CC between the model‐predicted and actual AEC‐derived values in the testing set were calculated. All the model training was performed using the Statistics and Machine Learning Toolbox (MATLAB, MathWorks, Massachusetts, USA).

**TABLE 3:** The training and testing datasets for patients without metal implants of group B. Data are expressed as mean ± standard deviation. Note that no significant difference was noted between the training and testing sets.

Group B	Age (years)	Weight (kg)	Height (cm)	BMI (kg/m^2^)	W/H (kg/m)	kVp	mAs	REX
Training set (M/F = 105/87)	58.1 ± 16.8	63.1 ± 12.8	161.6 ± 8.7	24.1 ± 3.9	0.39 ± 0.07	85.0 ± 0.0	30.0 ± 12.8	549.0 ± 68.9
Testing set (M/F = 23/18)	57.3 ± 17.4	62.0 ± 11.9	161.2 ± 8.3	23.8 ± 4.0	0.38 ± 0.07	85.0 ± 0.0	29.3 ± 11.1	543.0 ± 94.5

Abbreviations: BMI = body mass index; H = height; kVp = peak kilovoltage; mAs = milliampere second; REX = reached exposure; W = weight.

After constructing the model, the mean REX value of 716.3, derived from the 563 patients without metal implants in group A and corresponding to an average individual with a height of 162.4 cm and a weight of 67 kg, was incorporated into the model. The model with REX = 716.3 was then applied to the 56 patients with metal implants in group A to determine an appropriate mAs setting that ensures consistent image quality. After transfer learning, the mean REX value of 547.9, derived from the 234 patients without metal implants, was incorporated into the model to predict mAs for the 89 patients with metal implants in group B. Finally, the predicted mAs from the model and the actual mAs from the AEC technique for those patients with metal implants in the two groups were compared to assess the potential for reducing overexposure.

### 2.5 Statistical analysis

The demographic data of patients with and without metal implants, along with the data from the training and testing sets, were analyzed using a two‐sample *t*‐test with Bonferroni correction for multiple comparisons because these data were approximately normal distributed with equal variance. The CC, MAE, nRMSE, and R^2^ were compared to understand the performance of all ML models with 10‐fold cross validation using Mann–Whitney *U* test because of small number of measurements. The REX and mAs values obtained from the phantom experiment with and without a metal plate, along with the values at three different locations, were analyzed using analysis of variance (ANOVA) followed by post‐hoc Mann–Whitney *U* test. In the patients with metal implants, the mAs values predicted from the ML model and derived from the AEC technique were compared using a paired *t*‐test. The statistical analysis was performed using SPSS Version 20 (IBM SPSS statistics, USA). A *p*‐value < 0.05 was considered statistically significant.

## 3 RESULTS

In the phantom study, the results demonstrated that the mean REX and mAs values were 767.0 ± 9.1 and 59.8 ± 0.3, respectively, during KUB radiography. However, when a metal plate was placed on the phantom at the center, 5 cm away from the center, and the edge of FOV, the REX and mAs values were significantly increased to 1694.5 ± 17.5 and 131.6 ± 0.3, 1036.3 ± 8.4 and 80.6 ± 0.3, and 852.7 ± 4.1 and 67.1 ± 0.2, respectively, compared to those measured without a metal plate (up to 2.2‐fold increase in mAs). In addition, the ANOVA analysis demonstrated that REX and mAs values significantly varied with the locations of metal plate during KUB radiography.

In subsequent patient study, the demographic data in group A showed that patients with metal implants had significantly higher REX and mAs values than those without metal implants during KUB radiography, but no significant difference was noted in body height, weight, W/H, and BMI between them. Moreover, the comparison of five ML models demonstrated that ElmanNet ANN model outperformed other algorithms with the highest CC of 0.791 ± 0.007, *R* ^2^ of 0.6193 ± 0.010 and the lowest nRMSE of 0.285 ± 0.004 and MAE of 15.909 ± 0.309 in predicting the mAs for KUB radiography, as shown in Table 4.

**TABLE 4:** The comparisons of CC, MAE, nRMSE, and R^2^ between ML models for the 563 patients without metal implants of group A. Data are expressed as mean ± standard deviation. Asterisks (*) indicate statistical significance (*p* < 0.05) between the ElmanNet ANN and other ML models.

	Datasets	Gaussian SVM	RF	ElmanNet ANN	BAG	LASSO
CC	Training	0.9202 ± 0.002*	0.936 ± 0.001*	0.891 ± 0.003*	0.936 ± 0.001*	0.870 ± 0.001*
	Testing	0.730 ± 0.001*	0.731 ± 0.004*	0.791 ± 0.007*	0.734 ± 0.004*	0.780 ± 0.001
MAE	Training	8.957 ± 0.126*	9.007 ± 0.066*	11.260 ± 0.122*	8.997 ± 0.046*	12.468 ± 0.075
	Testing	18.348 ± 0.056*	18.253 ± 0.138*	15.909 ± 0.309*	18.182 ± 0.149*	16.377 ± 0.045
nRMSE	Training	0.167 ± 0.002*	0.155 ± 0.001*	0.193 ± 0.002*	0.155 ± 0.001*	0.210 ± 0.001*
	Testing	0.318 ± 0.001*	0.316 ± 0.002*	0.285 ± 0.004*	0.314 ± 0.002*	0.294 ± 0.001*
*R* 2	Training	0.845 ± 0.004*	0.868 ± 0.002*	0.795 ± 0.005*	0.867 ± 0.002*	0.756 ± 0.002*
	Testing	0.523 ± 0.002*	0.532 ± 0.005*	0.6193 ± 0.010*	0.535 ± 0.006*	0.604 ± 0.002*

Abbreviations: ANN = artificial neural network; BAG = bagging; CC = correlation coefficient; LASSO = least absolute shrinkage and selection operator; MAE = mean absolute error; nRMSE = normalized root mean squared error;RF = random forest; SVM = support vector machine.

**FIGURE 2:**
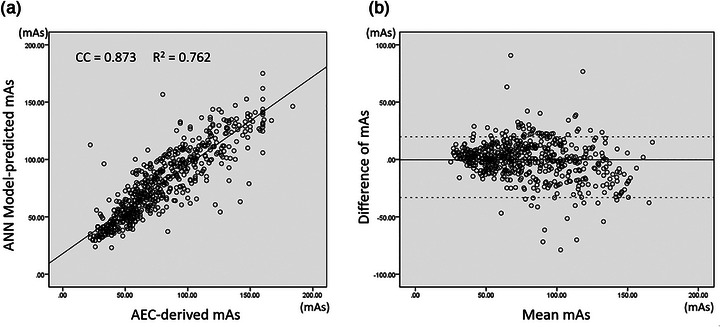
(a) The scatter plot and (b) Bland–Altman plot of ANN model‐predicted and actual AEC‐derived mAs in the 563 patients without metal implants with CC = 0.873 (*p* < 0.000) and *R* ^2^ = 0.762 in group A.

Afterward, the ElmanNet ANN model was then employed to predict the appropriate mAs for KUB radiography. The association and Bland–Altman plot between the model‐predicted and AEC‐derived mAs values for the 563 patients without metal implants of group A are shown in Figure 2.

In external validation based on transfer learning, the results showed that the ElmanNet ANN model achieved CC of 0.837 ± 0.051, nRMSE of 0.246 ± 0.005, MAE of 10.734 ± 0.582, and *R* ^2^ of 0.823 ± 0.007 in predicting the mAs for the testing set of group B. It was observed that the CC and R^2^ values were significantly higher in group B compared to those in group A, while the MAE and nRMSE values were significantly lower.

**FIGURE 3:**
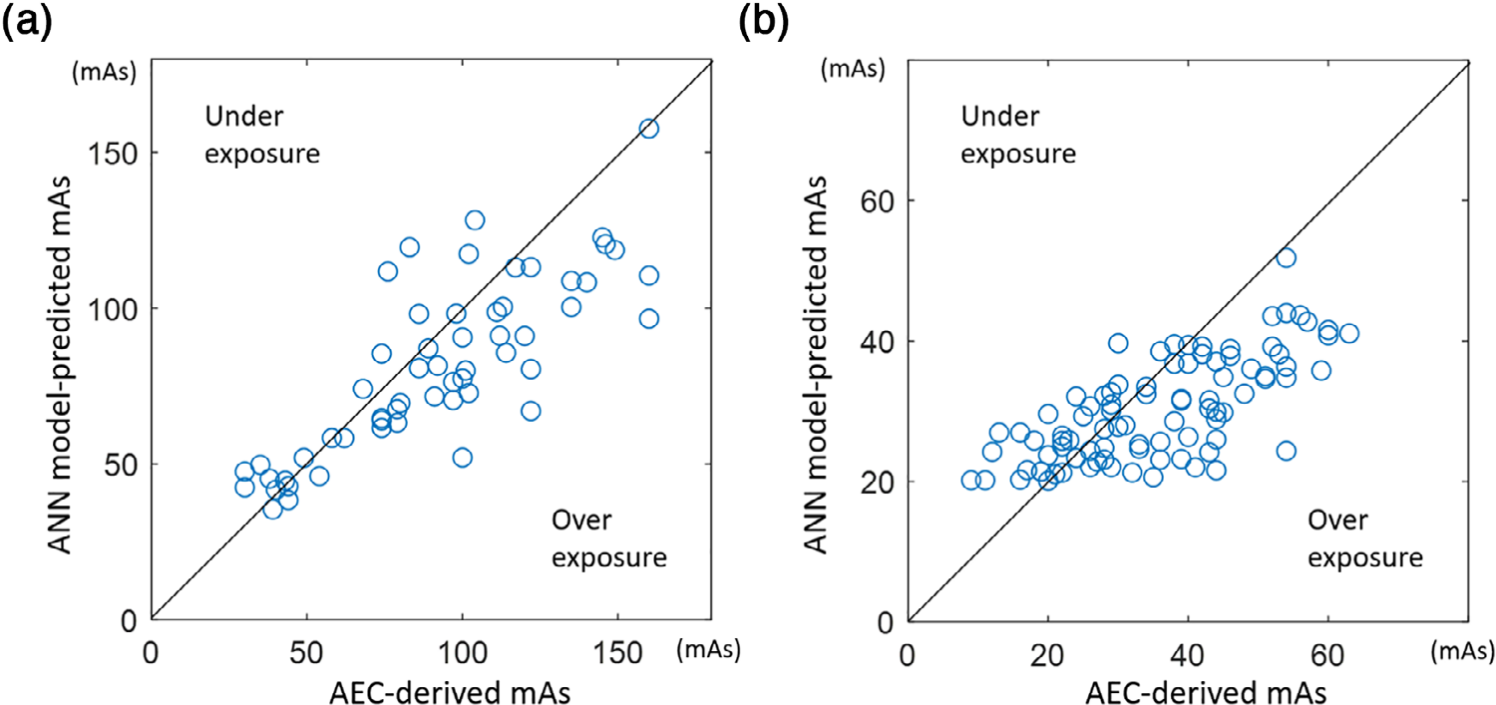
The scatter plots of ANN model‐predicted and actual AEC‐derived mAs in (a) the 56 patients with metal implants of group A and (b) 89 patients with metal implants of group B.

For patient groups A and B, the ElmanNet ANN model with the average REX = 716.3 and 547.9 of patients without metal implants was further employed to predict the appropriate mAs for the 56 and 89 patients with metal implants during KUB radiography, respectively. The ANN model‐predicted and AEC‐derived mAs values were significantly different (81.3 ± 24.0 vs. 92.1 ± 35.6) in the group A and (30.3 ± 7.2 vs. 35.6 ± 13.2) in the group B. The association between the model‐predicted and AEC‐derived mAs values in patients with metal implants is shown in Figure 3.

**FIGURE 4:**
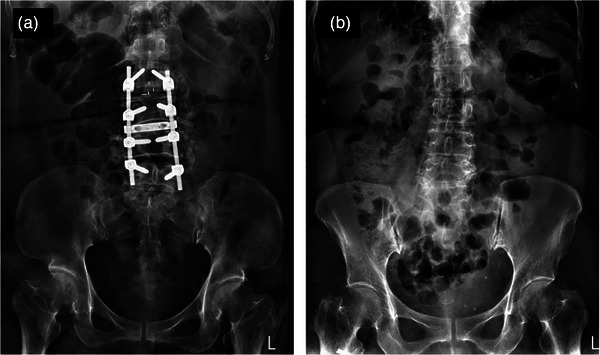
The KUB radiographs demonstrating the overexposure in a female patient with metal implant at the lumbar spine, (a) exposed at 84 kVp and 160 mAs, and normal exposure in another BMI‐matched female patient without metal implant, (b) exposed at 80 kVp and 70 mAs.

A female patient with a BMI of 33.3 (kg/m^2^) had metal implant in the lumbar spine and was exposed at 84 kVp and 160 mAs under AEC technique, resulting in an REX = 890. After performing the ElmanNet ANN model, the predicted mAs was reduced to 95.5 (about 40.3% reduction) for this patient, as shown in Figure 4a. For comparison, another female patient with a BMI of 33.3 (kg/m^2^) who did not have metal implant was exposed at 80 kVp and 70 mAs using the AEC technique, resulting in an REX = 719. After performing the ElmanNet ANN model, the predicted mAs was 96.6 for this patient, as shown in Figure 4b.

**FIGURE 5:**
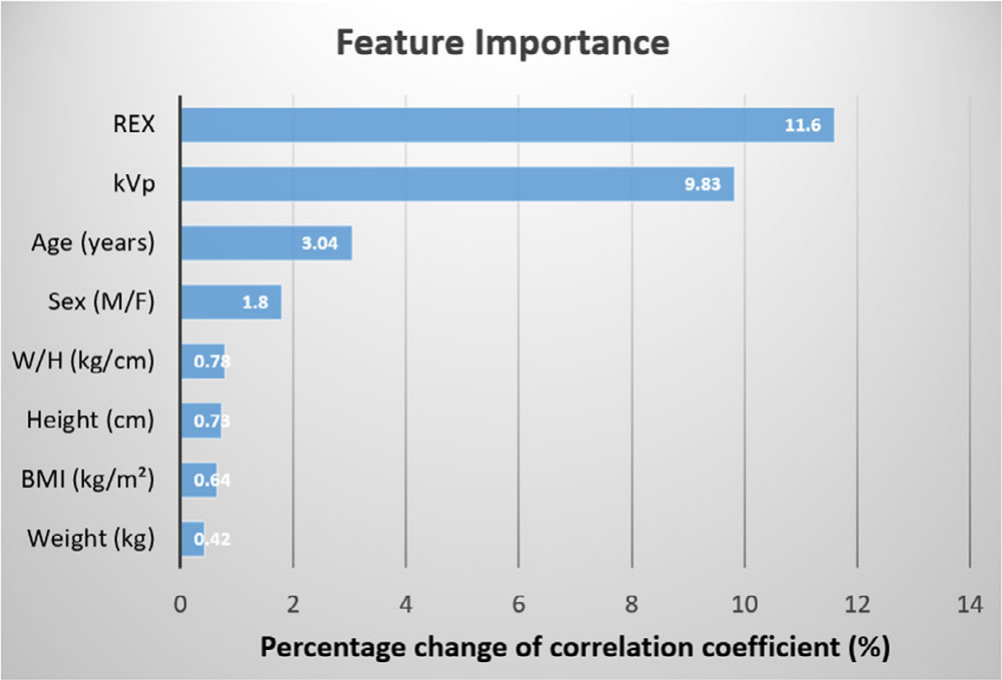
The feature importance of the ANN prediction model. BMI  =  body mass index; H  =  height; kVp = peak kilovoltage; REX  =  reached exposure; W  =  weight.

Moreover, the feature importance test of the ElmanNet ANN model showed that the REX and kVp played the most influential roles in predicting mAs for KUB radiography, as shown in Figure 5.

## 4 DISCUSSION

The present study conducted ML approach to establish a prediction model suitable for reducing overexposure in patients with metal implants undergoing KUB radiography, and had following main findings. First, the phantom experiment demonstrated that the existence of metal objects within FOV could lead to overexposure in KUB radiography. Second, the patients with metal implants had significantly higher mAs values than those without metal implants. Third, the ML approach built the ElmanNet ANN model that outperformed other ML models in predicting mAs for patients without metal implants. In addition, the external validation showed that the ElmanNet ANN model achieved similar performance in the external testing set. Finally, the proposed ElmanNet ANN model with a target exposure indicator could predict appropriate mAs that reduced overexposure for patients with metal implants in KUB radiography.

In the phantom experiment, this study employed an anthropomorphic phantom to illustrate the overexposure resulting from metal implants. By placing the metal plate on the phantom at the center, 5 cm away from the center, and the edge of FOV, the results demonstrated that mAs values significantly higher than those without a metal plate. The findings indicated that the overexposure was more significant when the metal plate was positioned at the center during KUB radiography, where AEC sensors located. Consistently, the REX values at center, 5 cm away, and the edge of FOV were found to significantly higher than those without a metal plate. The findings indicated that a higher mAs could lead to a higher REX value. Although the metal plate used in this study was different in size, shape, and materials from the actual orthopedic metal implants, the experiments provided evidence that when a metal object was within the FOV, both the mAs and REX values were significantly increased by using the AEC technique.

In the patient study, the comparison of demographic data also revealed that patients with metal implants exhibited significantly higher mAs and REX values compared to those without implants in both groups. The findings indicated that AEC technique may lead to overexposure in patients with metal implants. Therefore, the present study built the mAs prediction model exclusively using the data of patients without metal implants. By comparing different ML models for patients without metal implants, the results demonstrated that ElmanNet ANN model effectively predicted appropriate mAs settings in KUB radiography. Moreover, the external validation based on transfer learning further demonstrated that the ElmanNet ANN model could achieve similar performance in the testing set of the external dataset from a different hospital. These findings indicated that the proposed ElmanNet ANN model performed with good generalizability, suitable for predicting mAs for patients undergoing KUB radiography. It was also noted that the ElmanNet ANN in group B achieved better metrics than group A, suggesting that a fixed kVp led to higher prediction accuracy for mAs. Finally, the built ElmanNet ANN model was utilized to estimate appropriate mAs for patients with metal implants in both groups, and the results demonstrated that the ANN model‐predicted mAs values were significantly lower than actual AEC‐derived mAs values in patients with metal implants. These results indicate that the proposed ElmanNet ANN model not only accurately estimated the appropriate mAs for patients without metal implants but also mitigated overexposure for patients with metal implants prior to KUB radiography. In line with the previous ML chest radiography,^23^ the present study also demonstrated that ElmanNet ANN model outperformed other ML models, suggesting that the ElmanNet ANN model was suitable for predicting mAs in both KUB and chest radiography. For future applications, the established ML model could be implemented on the x‐ray control panel to predict appropriate mAs by retrieving patients’ physiological information from the PACS system prior to radiography. Additionally, the prediction model could be utilized to predict the upper mAs limit under the use of AEC technique, avoiding possible overexposure in patients with metal implants.

Furthermore, the importance permutation test revealed that the REX and kVp values were the two most significant features in predicting suitable mAs for KUB radiography. It is known that REX serves as an indicator of physical image quality, it reflects the quantity of x‐ray photons that reach the detector. Since the predicted mAs represents the amount of x‐ray photons generated by the x‐ray tube, it is anticipated that mAs is closely linked to image quality during radiography. Additionally, kVp indicates the energy of x‐ray photons, with higher kVp resulting in a greater number of higher‐energy x‐ray photons. In clinical practice, when a patient has a larger body size, a higher kVp is typically selected to produce more energetic x‐ray photons capable of penetrating thicker body parts. Alternatively, radiographers can adjust the mAs using the traditional 15% Rule to compensate for x‐ray exposure.^25^ Specifically, if the kVp is reduced by 15%, the mAs must be doubled to keep consistent exposure. As a results, both REX and kVp are crucial predictors in predicting the appropriate mAs during radiographic procedures.

This study has several limitations that deserve attention. First, it focused exclusively on subjects who underwent KUB radiography, limiting the applicability of the prediction model to mAs estimation within this specific radiographic procedure. Second, the iron plate had different geometry and attenuation from actual orthopedic metal implants, so the increased mAs and REX may differ from using real orthopedic metal implants. Third, the abdominal thickness was not recorded in routine physiological examination in the hospital. Further research will be needed to assess how abdominal thickness influences the accuracy of the prediction model. Fourth, the present study did not perform the dose‐image quality analysis for the patients before and after using predicted mAs, due to the retrospective study. In contrast, the present study integrated the target REX into the ElmanNet ANN model to predict an appropriate mAs that may produce a radiograph with consistent physical image quality. Further investigation will be needed to quantify the image quality using model‐predicted mAs. Fifth, the small number of patients with metal implants may lead to less statistical power. Lastly, the prediction model was developed and trained specifically for patients aged 18 years and older, making it inappropriate for younger patients undergoing KUB radiography.

## 5 CONCLUSIONS

This study utilized ML approach to construct models in predicting suitable mAs values for patients before undergoing KUB radiography. Our results clearly demonstrated that the ElmanNet ANN model outperformed the other ML models in predicting appropriate mAs values for patients without metal implants. By utilizing the prediction model, the mAs can be significantly reduced for patients with metal implants during KUB radiography. Therefore, we concluded that the ElmanNet ANN model is suitable for predicting appropriate mAs values and reducing overexposure for patients with metal implants prior to undergoing KUB radiography.

## AUTHOR CONTRIBUTIONS

**Wen‐Xuan Chen**: Conceptualization; methodology; validation; formal analysis; investigation; data curation; writing—original draft preparation. **Jen‐Pei Su**: Conceptualization; investigation; data curation. **Shih‐Hua Huang**: investigation; data curation. **Sin‐Rong Huang**: investigation; data curation. **Ming‐Chung Chou**: Conceptualization; methodology; software;validation; investigation; resources; writing—review and editing; supervision; project administration; funding acquisition. All authors have read and agreed to the published version of the manuscript.

## CONFLICT OF INTEREST STATEMENT

The authors declare no conflicts of interest.

## ETHICS STATEMENT

The local institutional review board of Kaohsiung Medical University Hospital (protocol code: KMUHIRB‐E(I)‐20200459) and E‐Da Hospital (protocol code: EMRP‐109‐165) approved this study. The study was conducted in accordance with the Helsinki Declaration, and the need to obtain the informed consent was waived due to the retrospective nature of the study.

## Data Availability

The datasets used and/or analyzed during the current study available from the corresponding author on reasonable request.
